# *TFF1* hypermethylation and decreased expression in esophageal squamous cell carcinoma and histologically normal tumor surrounding esophageal cells

**DOI:** 10.1186/s13148-017-0429-0

**Published:** 2017-12-20

**Authors:** Isabela Martins Gonzaga, Sheila Coelho Soares Lima, Marina Chianello Nicolau, Pedro Nicolau-Neto, Nathalia Meireles da Costa, Tatiana de Almeida Simão, Hector Hernandez-Vargas, Zdenko Herceg, Luis Felipe Ribeiro Pinto

**Affiliations:** 1 Programa de Carcinogênese Molecular, Instituto Nacional de Câncer, Coordenação de Pesquisa, Rua André Cavalcanti, 37–6° andar, Bairro de Fátima, Rio de Janeiro, Rio de Janeiro CEP: 20231-050 Brazil; 2 Departamento de Bioquímica, Instituto de Biologia Roberto Alcantara Gomes, Universidade do Estado do Rio de Janeiro, Av. 28 de Setembro 87 fundos, Vila Isabel, Rio de Janeiro, CEP: 20551-013 Brazil; 3 Epigenetics Group, Section of Mechanisms of Carcinogenesis, International Agency for Research on Cancer, 150 Cours Albert Thomas, 69372, CEDEX 08 Lyon, France

**Keywords:** Esophageal squamous cell carcinoma, Esophageal cancer, Trefoil factors, TFF1, Biomarker, DNA methylation

## Abstract

**Background:**

Esophageal squamous cell carcinoma (ESCC) is one of the 10 most incident cancer types in the world, and it is mainly associated with tobacco and alcohol consumption. ESCC mortality rates stand very close to its incidence, which is a direct consequence of a late diagnosis and an inefficient treatment. Although this scenery is quite alarming, the major molecular alterations that drive this carcinogenesis process remain unclear. We have previously shown through the first ESCC methylome analysis that *TFF1* promoter is frequently hypermethylated in ESCC. Here, to evaluate *TFF1* methylation as a potential biomarker of early ESCC diagnosis, we investigated the status of *TFF1* promoter methylation and its expression in ESSC and histologically normal tumor surrounding tissue of ESCC patients in comparison to healthy esophagus of non-cancer individuals.

**Results:**

Analysis of *TFF1* promoter methylation, and gene and protein expression in 65 ESCC patients and 88 controls revealed that *TFF1* methylation levels were already increased in histologically normal tumor surrounding tissue of ESCC patients when compared to healthy esophagus of non-cancer individuals. This increase in DNA methylation was followed by the reduction of *TFF1* mRNA expression. Interestingly, *TFF1* expression was capable of distinguishing tumor surrounding normal tissue from normal mucosa of healthy individuals with 92% accuracy. In addition, TFF1 protein was undetectable both in tumor and surrounding mucosa by immunohistochemistry, while submucosa glands of the healthy esophagus showed positive staining. Furthermore, treatment of TE-1 and TE-13 ESCC cell lines with decitabine led to a reduction of promoter methylation and consequent upregulation of TFF1 gene and protein expression. Finally, using TCGA data we showed that *TFF1* loss is observed in ESCC, but not in esophageal adenocarcinoma, highlighting the different molecular mechanisms involved in the development of each histological subtype of esophageal cancer.

**Conclusions:**

This study shows that *TFF1* expression is silenced in early phases of ESCC development, which seems to be mediated at least in part by promoter hypermethylation, and provides the basis for the use of *TFF1* expression as a potential biomarker for early ESCC detection.

**Electronic supplementary material:**

The online version of this article (10.1186/s13148-017-0429-0) contains supplementary material, which is available to authorized users.

## Background

Esophageal cancer (EC) is one of the most incident tumors worldwide. The two main EC histological subtypes are esophageal adenocarcinoma (EAC) and esophageal squamous cell carcinoma (ESCC), which show marked differences in relation to associated risk factors and molecular mechanisms of development [1]. The latter is the most frequent histological subtype and, in addition to its high incidence, ESCC ranks as fifth in cancer mortality among men, with a 5-year overall survival below 15% [2, 3]. The poor prognosis of ESCC patients is a consequence of late diagnosis and poor response to treatment [4, 5]. Therefore, there is an urgent need to develop biomarkers for early ESCC diagnosis and risk stratification.

The first ESCC methylome analysis revealed that *TFF1* promoter hypermethylation was a common alteration present in these tumors [6]. TFF1 is one of the three Trefoil factors (TFF), protease-resistant peptides, which contain a conserved three loop domain, designated as TFF domain [7–9]. The main function of these peptides is related to the maintenance of gastrointestinal mucosa integrity, acting in the protection and recovery of damaged epithelium [10]. However, alterations in the expression of these peptides have been described in several types of cancer, suggesting their role in the carcinogenesis process. *TFF3* overexpression is frequently observed in pancreatic [11], hepatocellular [12], colon [13], and gastric cancer [14], being correlated with tumor grade and a poor prognosis [15, 16]. In contrast, gastric cancer usually displays reduced *TFF1* expression levels and *TFF1*-knockout mice are prone to develop gastric carcinomas [17], consistent with the notion that the downregulation of TFF1 may play an important role in the development of this cancer type.

In the present study, we aimed to investigate the expression profile of TFF1, its regulation by gene promoter methylation, and its potential to be used as an earlier diagnostic biomarker in ESCC.

## Methods

### Human samples

Sixty-five patients with a confirmed histological diagnosis of ESCC who had not undergone chemo or radiotherapy were recruited between 1997 and 2015 from two hospitals in Brazil: Instituto Nacional do Câncer (INCA, Rio de Janeiro) and Hospital de Clínicas de Porto Alegre (HCPA-UFRGS, Porto Alegre). Tumor and histologically normal adjacent mucosa were obtained either as formalin-fixed paraffin embedded (FFPE) or fresh snap frozen tissue. Patients’ information was collected from their medical records or from a standardized questionnaire. In addition to patients, 88 subjects without cancer and with no alterations in the esophagus who were submitted to endoscopy for other reasons at Hospital Universitário Pedro Ernesto (HUPE-UERJ, Rio de Janeiro) were also included in the study (control group). From these individuals, biopsies were collected as FFPE or fresh snap frozen samples from the middle third of the esophagus. The controls also answered the standardized questionnaire and all individuals signed a consent form. The project was approved by the Ethic Committees of all institutions involved.

The characteristics of ESSC patients and healthy individuals included in this study are summarized in Table 1. The median age of ESCC patients was 59 years, ranging from 39 to 78 years, with most of the patients being male (78%), drinkers (66%), and smokers (81%). Tumors were most often located in the middle third of the esophagus (72%), with a higher prevalence of advanced stage of the disease (69%) and a high mortality rate (69%; median survival of 12 months). Among healthy individuals, the median age was 58 years, mostly females (67%), never or non-current drinkers (56%) and smokers (85%) (Table 1).

**Table 1:** Characteristics of the individuals included in the study

	Healthy subjects*	ESCC patients*
Gender		
Male	27 (33%)	50 (78%)
Female	55 (67%)	14 (22%)
Age (median and range)	58 (20–85)	59 (39–78)
Tobacco smoking		
Never smokers	48 (60%)	1 (3%)
Former smokers	13 (25%)	6 (17%)
Current smokers	20 (15%)	29 (81%)
Alcohol drinking		
Never drinkers	41 (50%)	8 (25%)
Former drinkers	5 (6%)	3 (9%)
Current drinkers	36 (44%)	21 (66%)
Biopsy localization		
Middle third	88 (100%)	
Tumor localization		
Upper third		2 (3%)
Upper-middle thirds		4 (6%)
Middle third		33 (52%)
Middle-lower thirds		9 (14%)
Lower third		15 (24%)
Tumor differentiation		
in situ		1 (2%)
Well		0 (0%)
Moderate		49 (79%)
Poor		12 (19%)
T (TNM)		
Ti		1 (2%)
T1		8 (14%)
T2		13 (22%)
T3		28 (47%)
T4		9 (15%)
N (TNM)		
N0		26 (52%)
N1		24 (48%)
M (TNM)		
M0		26 (76%)
M1		8 (24%)
Tumor stage		
0		1 (3%)
I		3 (9%)
II		7 (20%)
III		16 (46%)
IV		8 (23%)

*number of patients and controls may vary due to missing data

### Cell lines and treatment with 5-Aza-2-deoxycytidine (decitabine)

The ESCC cell lines TE-1 and TE-13 were gently donated by the Dr. Pierre Hainaut from the International Agency for Research on Cancer (IARC). Cells were kept at 37 °C, under 5% CO_2_ in Dulbecco’s modified Eagle’s medium (DMEM) (Gibco) or RPMI 1640 (Gibco) supplemented with 10% fetal bovine serum, 100 units/mL penicillin, 0.1 mg/mL streptomycin, and L-glutamine. To perform decitabine (Sigma) treatment, 1.5 × 10^5^ cells were seeded in 6-well plates. After 24 h, decitabine was added to the culture medium in a final concentration of 2.5 μM and the medium was replaced after 48 h of incubation. The cells were collected for DNA, RNA, and protein isolation after 72 h of treatment. In the controls, DMSO, the solvent of decitabine, was added. A total of three independent experiments in triplicates were performed. The authenticity of TE-1 and TE-13 cells was confirmed by Powerplex 18D STR System (Promega) and was routinely tested for Mycoplasma using Mycosensor PCR assay kit (Agilent).

### DNA and RNA isolation

DNA and RNA isolation from frozen samples and cell lines were performed using the DNeasy Blood & Tissue Kit (Qiagen®, Germany) and RNeasy Mini Kit (Qiagen®, Germany), respectively, according to the manufacturer’s protocols. The concentration and purity of the nucleic acids were measured by spectrophotometry.

### RT-qPCR

In order to synthesize cDNA, 500 ng of total RNA were used in reverse transcription (RT) reactions using the SuperScript II Reverse Transcriptase (Life Technologies™, USA). For the evaluation of *TFF1* and *GAPDH* expression, pairs of oligonucleotides spanning exon-exon junctions were designed: *TFF1* Forward: GACAGAGACGTGTACAGTGGC; *TFF1* Reverse: CGATGGTATTAGGATAGAAGCA; *GAPDH* Forward: 5′-CAACAGCCTCAAGATCATCAGCAA-3′; *GAPDH* Reverse: 5′-AGTGATGGCATGGACTGTGGTCAT-3′. The qPCR was performed in the thermocycler Corbett (Qiagen®, Germany). Each reaction consisted of 5.0 μL of QuantiFast SYBR® Green Master Mix (Qiagen®, Germany), 10 pmols of each oligonucleotide, 1 μL of cDNA (diluted 10×), and sterile deionized water to complete the final volume of 10 μL. The amplification reaction was performed as follows: 5 min of predenaturation at 95 °C followed by 40 cycles of denaturation for 5 s at 95 °C and an annealing and extension step for 10 s at 60 °C. After the reaction, *TFF1* mRNA expression was normalized by the expression of *GAPDH*. The mRNA relative quantification was calculated by the ΔCt method [18]. Gene expression analyses were performed in a total of 33 healthy esophageal samples, and 24 pairs (normal-appearing adjacent mucosa and tumors) from ESCC patients.

### Pyrosequencing

The methylation status in the selected CpG site was examined by pyrosequencing essentially as described previously [6, 19]. Briefly, a total of 1 μg of genomic DNA from human samples and cell lines treated or not with decitabine was modified with sodium bisulfite using the EZ DNA Methylation-Gold Kit (Zymo Research®, USA). The DNA was eluted to reach a final concentration of 25 ng/μL. To assess *TFF1* promoter methylation status, PCR was performed with Platinum®Taq DNA Polymerase (Life Technologies™), using the following oligonucleotides: F: 5′-GGTTGTTAGAGTTGGTTGTGG-3′; R: 5′-biotin-CTAAATCTCAAATCCCTCAACC-3′. The PCR was performed as follows: 5 min of predenaturation at 95 °C, 50 cycles consisting of denaturation at 95 °C for 30 s, annealing at 56 °C for 30 s and extension at 72 °C for 30 s, followed by a final extension at 72 °C for 10 min. PCR products were visualized in 2% agarose gel electrophoresis. To quantify the percentage of methylated cytosines, PCR products were sequenced using a pyrosequencing system (PSQTM 96MA, Qiagen®, Germany) and the sequencing oligonucleotide 5′-GAAGGATTTGTTGATAGA-3′, in accordance with the manufacturer’s protocol (Qiagen®, Germany). This method treats each individual CpG site as a C/T polymorphism and generates quantitative data for the relative proportion of the methylated versus the unmethylated allele. The target CpG was evaluated by converting the resulting pyrograms into numerical values for peak heights. *TFF1* promoter methylation analyses were performed in a total of 33 healthy esophageal samples, and 58 pairs (normal-appearing adjacent mucosa and tumors) from ESCC patients.

### Protein isolation and western blotting

Protein extraction was performed by washing cells twice in ice-cold PBS and subsequently lysing them by using RIPA-like buffer (250 mM NaCl, 50 mM TRIS-HCl pH 7.4, 0.1% SDS, 2 mM DTT, and 0.5% NP-40), containing protease inhibitors (Complete-Mini, Roche). Bradford assay (Bio-Rad) was employed in order to determine protein concentration, using bovine serum albumin as standard. A total of 70 μg of proteins from each experimental condition were resolved onto a 8.0% SDS-PAGE, were transferred a to nitrocellulose-membrane, by using the iBlot® Dry Blotting System, according to manufacturer’s instructions (Invitrogen), and were probed with primary antibodies anti-TFF1 (1:250, Abcam ab92377) and anti-lamin A/C (1:1000, Cell Signaling #4777), used as loading control. Membranes were then incubated with the horseradish peroxidase-conjugated secondary antibody (1:10,000), and detection was performed with enhanced chemiluminescence (ECL Kit, Amersham).

### Immunohistochemistry

Immunohistochemistry was performed on paraffin sections of 40 ESCC cases and 24 controls. For antigen retrieval, sections were incubated in a water bath while submerged in citrate buffer, pH 6.0, for 40 min at 98 °C. Sections with 3 μm were then incubated in 3% hydrogen peroxide for 20 min and Protein Block solution for 30 min (Dako®, Denmark) before the incubation with the primary antibody against TFF1 (Abcam®–ab92377), overnight at 4 °C. Sections were then washed and covered with biotinylated secondary antibody for 30 min at room temperature followed by incubation in peroxidase-conjugated streptavidin for 30 min. Detection was performed with the LSAB System (Dako®, Denmark), and the staining was carried out with diaminobenzidin (DAB, Dako®). Sections were counterstained with Harris’ hematoxylin. FFPE lung adenocarcinoma was used as positive control of TFF1 expression. In the negative control, the primary antibody was replaced with the antibody diluent solution.

### Analyses of *TFF1* expression and DNA methylation data deposited in The Cancer Genome Atlas (TCGA) database

Gene expression data from EC samples (*n* = 183), both esophageal adenocarcinoma (*n* = 88) and squamous cell carcinoma (*n* = 95), was downloaded from the public database cBioPortal for Cancer Genomics [20, 21], which provides visualization, analysis, and download of large-scale data sets deposited in The Cancer Genome Atlas (TCGA). The DNA methylation data from the same samples was downloaded using the web-based software Wanderer [22].

### Statistical analyses

All statistical analyses were performed using the GraphPad Prism 5 software (GraphPad Software, USA). Differences were considered statistically significant when *p* value < 0.05. Unpaired *t* test or Mann-Whitney test was applied when comparing two groups. When comparing three or more groups, one-way ANOVA or Kruskal-Wallis test and Dunn’s post test were used. With the intent to evaluate the use of *TFF1* expression as a marker to distinguish healthy esophagus from normal-appearing ESCC adjacent mucosa, a receiver operating characteristic (ROC) curve was plotted. Spearman test was used for correlation analyses.

## Results

### TFF1 expression and promoter methylation in healthy esophagus and ESCC

**Fig. 1:**
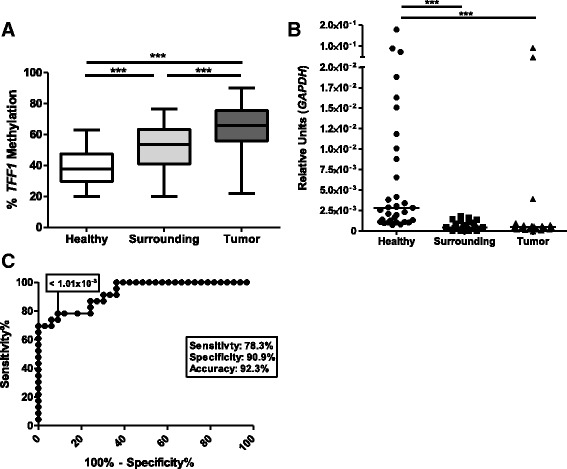
Molecular alterations of *TFF1* in ESCC. **a** *TFF1* promoter methylation status in tumor, normal-appearing surrounding tissue, and healthy esophageal mucosa assessed by pyrosequencing. **b** TFF1 mRNA expression in tumor, normal-appearing surrounding tissue, and healthy esophageal mucosa evaluated by RT-qPCR. **c** ROC curve of *TFF1* mRNA expression for discrimination of healthy esophagus and normal-appearing surrounding tissue from ESCC patients. **p < 0.01; ***p < 0.001

Initially, we assessed the methylation status of *TFF1* promoter in ESCC patients and subjects without cancer by pyrosequencing. *TFF1* promoter was hypermethylated in tumor and histologically normal tumor surrounding tissue in comparison with healthy esophagus (Fig. 1a). The promoter methylation median in healthy esophagus was 35.26%, while it was 53.4% for surrounding mucosa and 68.2% for tumor samples. We next analyzed *TFF1* expression levels in the same sample groups and found a lower expression of *TFF1* in ESCC and histologically normal surrounding tissue (median of 5 × 10^−4^ and 4.3 × 10^−4^, respectively) when compared with healthy esophageal epithelium (median of 2.8 × 10^−3^, Fig. 1a). Since the reduction of *TFF1* expression was already observed in histologically normal tumor surrounding cells, we evaluated if *TFF1* mRNA expression could distinguish esophageal mucosa from healthy individuals from histologically normal surrounding tissue from ESCC patients. Using a Receiver Operator Characteristic (ROC) curve, we showed that *TFF1* mRNA levels (1.01 × 10^−3^) are able to discriminate these two histologically normal tissue samples with an accuracy of 92.3%, a sensitivity of 78.3%, and a specificity of 90.9% (*p* < 0.0001, Fig. 1c). With a cut-off of 50.05%, *TFF1* promoter methylation levels were also capable of discriminating the two groups of samples (*p* < 0.0001), but with lower accuracy (78.03%) and sensitivity (67.24%) (data not shown). Taken together, our results suggest that a *TFF1* promoter methylation increase of less than 20% can result in a reduction of gene expression greater than 80% and total absence of protein expression, as observed when comparing healthy esophagus and non-tumor adjacent mucosa from ESCC patients.

**Fig. 2:**
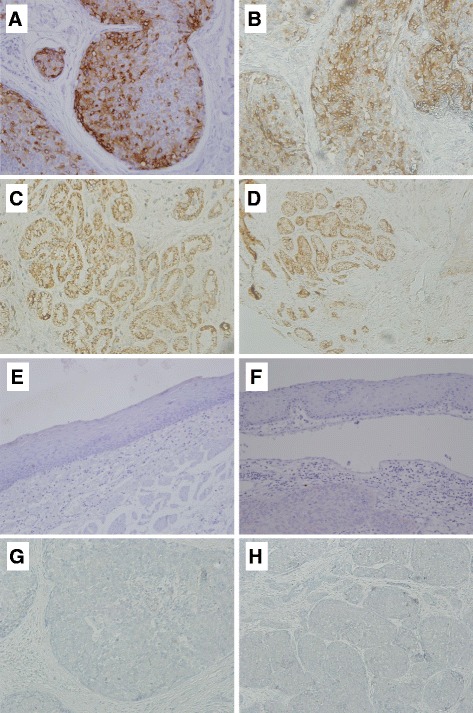
TFF1 protein expression in ESCC and healthy esophagus. Representative images of TFF1 immunostaining for each sample group are shown. **a**, **b** Lung adenocarcinoma, used as positive control (**a**, × 20 magnification, **b** × 40 magnification). **c**, **d** Healthy esophagus, cytoplasmatic expression of TFF1 was detected in submucosa glands of all samples evaluated (*n* = 22; C, × 20 magnification, **d**, × 10 magnification). **e**, **f** Normal-appearing surrounding mucosa from ESCC patients, all samples (*n* = 7) were negative for TFF1 staining (**e** and **f** × 20 magnification). **g**, **h** ESCC, all samples (*n* = 48) were negative for TFF1 staining (**g** and **h**, × 20 magnification)

TFF1 protein expression was evaluated by immunohistochemistry, with no staining being observed in tumors (*n* = 48) or surrounding tissue (*n* = 7) (Fig. 2e–h). TFF1 expression was only detected in healthy esophageal samples (*n* = 22), specifically in the cytoplasm of all submucosa glands (Fig. 2c, d), while epithelial cells showed no TFF1 expression. These findings are consistent with the hypothesis that methylation-dependent silencing of *TFF1* may occur in both ESCC and tumor adjacent normal-appearing mucosa.

Given that we observed variable *TFF1* mRNA expression levels among healthy individuals (Fig. 1b), we sought to evaluate whether this could be a consequence of exposure to risk factors. However, we found no association between *TFF1* expression levels or promoter methylation rate in normal esophageal cells and tobacco smoking or alcohol drinking, two well-recognized risk factors for this cancer type (Additional file 1: Table S1), suggesting that methylation-mediated silencing of *TFF1* is independent of these risk factors. The same was observed in ESCC samples, in which *TFF1* expression and methylation were not associated with tobacco or alcohol consumption (Additional file 2: Table S2).

Moreover, we assessed the possible association between tumor *TFF1* expression or methylation status and the clinicopathological characteristics of ESCC patients. No associations between *TFF1* expression or methylation rate and tumor differentiation, TNM, or tumor stage were observed (Additional file 2: Table S2).

### Regulation of TFF1 expression by DNA methylation in vitro

**Fig. 3:**
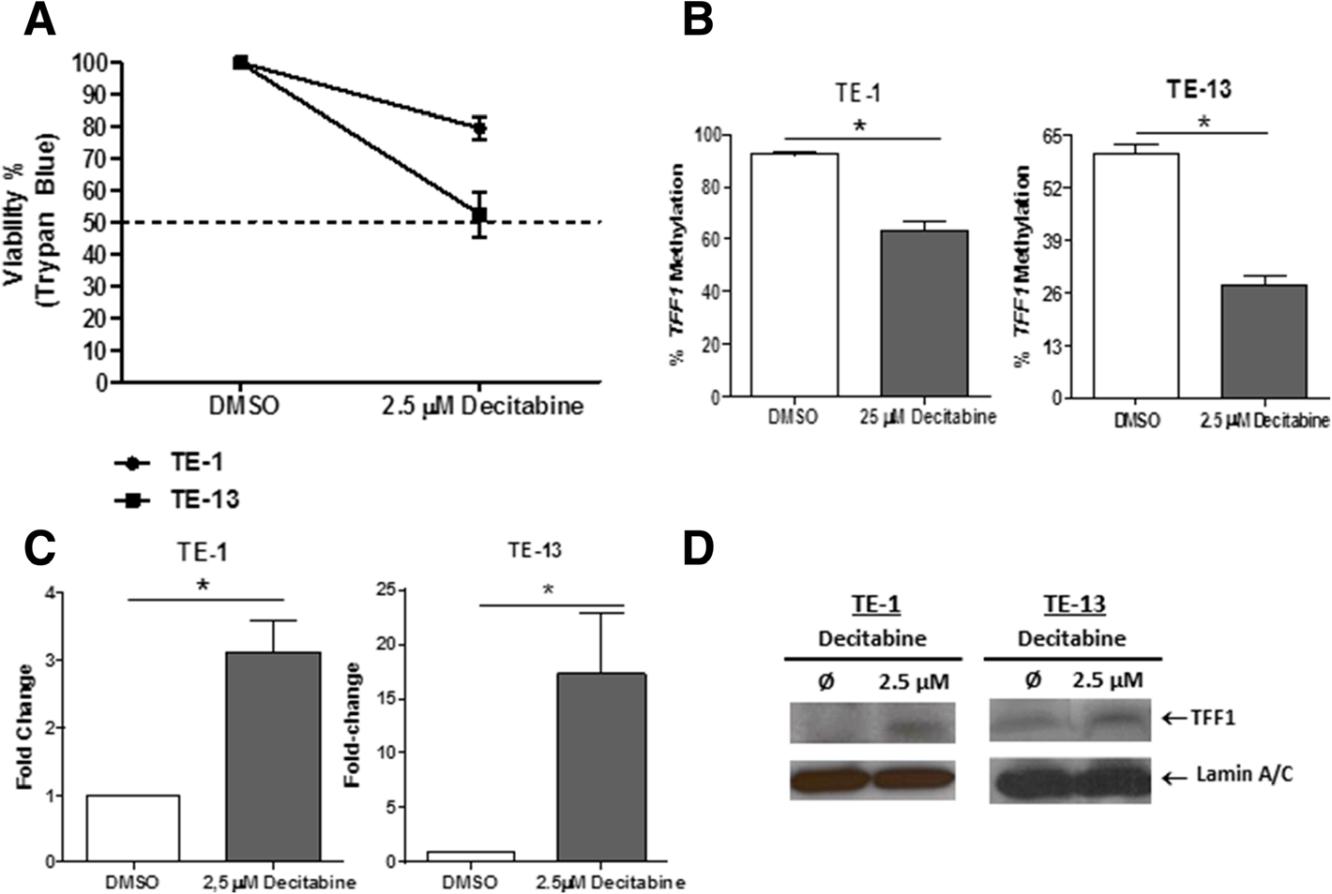
Promoter methylation contributes to the downregulation of *TFF1* mRNA and protein levels in vitro. **a** Cell viability, assessed by trypan blue staining, of TE-1 and TE-13 cells after treatment with DMSO or 2.5 μM of decitabine for 72 h. **b** *TFF1* promoter methylation status, assessed by pyrosequencing, in TE-1 and TE-13cells after treatment with DMSO or 2.5 μM of decitabine for 72 h. **c** *TFF1* mRNA expression levels, assessed by RT-qPCR, in TE-1 and TE-13cells after treatment with DMSO or 2.5 μM of decitabine for 72 h. **d** TFF1 protein expression, assessed by western blot, in TE-1 and TE-13cells after treatment with DMSO or 2.5 μM of decitabine for 72 h.*p < 0.05

To confirm the regulation of *TFF1* expression by its promoter methylation, we treated the ESCC-derived cell lines TE-1 and TE-13 with 2.5 μM decitabine (5-aza-2′-deoxycytidine, DNA methyltransferase inhibitor) for 72 h, resulting in more than 50% cell viability (Fig. 3a). Decitabine treatment resulted in a decrease of *TFF1* promoter methylation levels of approximately 30 and 60%, with a concomitant increase of *TFF1* expression of around 3- and 15-fold, for TE-1 and TE-13 cells, respectively (Fig. 3b, c). In addition, TFF1 protein expression was also induced in both cell lineages after decitabine treatment (Fig. 3d).

### TFF1 expression and promoter methylation in different esophageal cancer histological subtypes

**Fig. 4:**
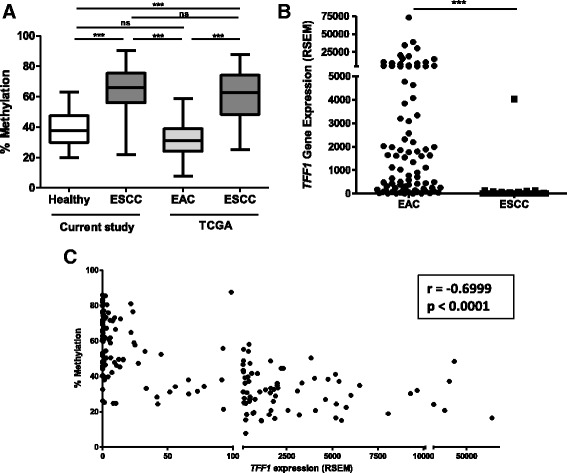
*TFF1* promoter methylation and mRNA expression differ in esophageal cancer histological subtypes. **a** TFF1 promoter methylation status in healthy esophagus and esophageal squamous cell carcinoma (ESCC) from the current study and in esophageal adenocarcinoma (EAC) and ESCC from the TCGA consortium. **b** TFF1 mRNA expression in EAC and ESCC, assessed by RNA-sequencing in the TCGA consortium. **c** Correlation analysis of *TFF1* promoter methylation and mRNA expression in EAC and ESCC in TCGA data. ***p < 0.001

With the purpose of evaluating whether the reduction of *TFF1* expression and augment of its promoter methylation are involved in esophageal carcinogenesis in general, independently of histological subtype, we evaluated these parameters in ESCC and esophageal adenocarcinoma (EAC) using the TCGA database. We observed a median methylation of *TFF1* promoter of 62.6% in ESCC, which is comparable to what was observed in the present study for Brazilian patients (65.9%) (Fig. 4a). We further found that *TFF1* promoter methylation was significantly lower in EAC (median of 31.15%) and comparable to our results for healthy individuals (median of 38.4%, Fig. 4a). Differences between the two EC histological subtypes were also observed when *TFF1* expression was evaluated, with EAC showing significant higher levels of *TFF1* mRNA, when compared with ESCC (Fig. 4b). Finally, a significant inverse correlation between *TFF1* expression and promoter methylation was observed for esophageal tumor samples (*r* = − 0.6999, *p* < 0.0001) (Fig. 4c). These results demonstrate that aberrant methylation-mediated silencing of *TFF1* is specific for ESCC.

## Discussion

In the present study, we showed that *TFF1* expression is silenced early in the development of ESCC, which is mediated at least in part by promoter hypermethylation, resulting in the complete absence of TFF1 protein. *TFF1* expression presents high accuracy in discriminating histologically normal ESCC surrounding tissue from esophageal mucosa from healthy individuals. Finally, we show that silencing of *TFF1* expression by promoter hypermethylation is a specific feature of ESCC, since the same profile was not observed in esophageal adenocarcinoma.

The mechanisms that lead to ESCC development are still largely unknown. This lack of knowledge directly reflects on the identification and application of biomarkers of early diagnosis, and consequently on a poor efficacy of the treatment. Interestingly, our data shows that *TFF1* expression levels are capable of discriminating esophageal mucosa from healthy individuals from histologically normal tumor surrounding tissue with 92.3% of accuracy. Since this molecular event precedes tissue morphological alterations, especially in groups at risk of developing this disease, epigenetic-silencing of *TFF1* has a potential for application in ESCC early diagnosis. Among these, patients with head and neck squamous cell carcinoma (HNSCC) present a higher chance of developing a second primary tumor in the esophagus (SPTE) [23]. In those cases, the overall survival rate remarkably decreases since the second primary ESCC is usually diagnosed at late stages [24]. The second primary ESCC tumors arise from the formation of the cancerization field, a phenomenon originally described by Slaughter [25], in which the squamous epithelium of the upper aerodigestive tract is altered in head and neck cancer patients. In this context, the validation of *TFF1* expression as a biomarker of early alterations in the esophageal mucosa in a larger study, including patients with HNSCC, is of utmost relevance and may not only enable an early diagnosis, but also indicate which patients are more likely to develop a SPTE.

TFF1 is a mucosa protector factor, which is upregulated upon injuries, participates in the maintenance of integrity of mucosa and is involved in stomach ontogenesis [26, 27]. In gastric cancer, the loss of *TFF1* can contribute to the induction of pro-inflammatory and anti-apoptotic genes through activation of NF-κB pathway [28]. *TFF1* is classified as a tumor suppressor gene in gastric and may have a similar role in other tumor types [29, 30]. For example, the absence of *TFF1* enhances the tumorigenic abilities of MCF7, a breast cancer cell line, in vitro and in vivo. Similarly, TFF1-KO also enhances tumor formation in ovary and lung using a 7,12-dimethylbenz[a]anthracene (DMBA)-induced carcinogenesis model [30]. Interestingly, previous studies have shown *TFF1* upregulation in Barrett’s esophagus, a premalignant condition which increases the risk of esophageal adenocarcinoma development. However, in EAC, our results showed that *TFF1* mRNA levels are similar to normal esophagus, confirming previous observations [31, 32]. This suggests that *TFF1* expression might be induced in response to the injury caused by the acid reflux, which is associated with Barrett’s esophagus, but ceases during the progression to EAC. Taken together, these results suggest that *TFF1* may have a role as a tumor suppressor in ESCC and not in EAC, although further studies are necessary to confirm this hypothesis.

It has been recently shown that EAC resembles gastric adenocarcinoma with respect to molecular alterations, while ESCC is closer to squamous carcinomas of the head and neck [33]. However, it is important to mention that EAC is closely related to gastric cancers with chromosomal instability and these two tumors clearly differ from gastric adenocarcinoma related to Epstein-Barr infection, microsatellite instability, and genomic stability. Since the studies that focused on *TFF1* deregulation in gastric cancer did not consider these molecular subgroups, it is difficult to establish whether *TFF1* loss would be a common feature in this cancer type or subgroup-specific. Furthermore, very few studies have evaluated TFF1 expression in head and neck cancer so far. In salivary gland tumors, a higher expression of TFF1, TFF2, and TFF3 was found [34], while in oral squamous cell carcinoma TFF2 and TFF3, but not TFF1, were downregulated with respect to healthy tissue [34]. However, based on so few studies, we cannot come to a conclusion on the role of TFF proteins, specifically TFF1, in head and neck cancer. Therefore, this apparent paradox should be further explored.

Our findings also showed that TFF1 mRNA and protein expression were increased after decitabine treatment in ESCC cell lines, while promoter methylation levels decreased. This supports the notion that the absence of TFF1 in the esophagus of ESCC patients is related to epigenetic mechanisms. The regulation of *TFF1* expression by promoter methylation has already been described in other tumors. Lack of *TFF1* expression was correlated with high promoter methylation levels in patients with gastric cancer, and in vitro analyses also demonstrated that gastric cancer cells exposed to demethylating agents show increased levels of *TFF1* expression [35–37]. In addition, treatment with 5-aza-2′-deoxycytidine induces *TFF1* expression in prostate cells that normally do not express this gene [38].

## Conclusions

In summary, the present study suggests the potential use of *TFF1* expression as a biomarker for early ESCC detection. We show, for the first time, a clear downregulation of *TFF1* gene and protein expression in ESCC and normal-appearing surrounding tissue when compared with healthy esophagus, which seems to be mediated at least in part by promoter hypermethylation. However, other mechanisms may be involved and should be explored in future studies. Finally, *TFF1* downregulation is likely to contribute to ESCC, but not EAC development.

## Additional files

Additional file 1: Table S1. Association between clinicopathological data and *TFF1* methylation and expression in healthy esophagus. (DOC 129 kb)

Additional file 2: Table S2. Association between clinicopathological data and *TFF1* expression in ESCC. (DOC 238 kb)

## Abbreviations

DAB: Diaminobenzidin
EAC: Esophageal adenocarcinoma
EC: Esophageal cancer
ESCC: Esophageal squamous cell carcinoma
FFPE: Formalin-fixed paraffin embedded
GAPDH: Glyceraldehyde-3-phosphate dehydrogenase
HNSCC: Head and neck squamous cell carcinoma
TCGA: The Cancer Genome Atlas
TFF: Trefoil factors
