# Supplementary material for: TFF1 hypermethylation and decreased expression in esophageal squamous cell carcinoma and histologically normal tumor surrounding esophageal cells
Source: Clin Epigenetics. 2017 Dec 20;9:130. doi: 10.1186/s13148-017-0429-0 (PMC5738900; doi:10.1186/s13148-017-0429-0)
Supplement: Supplementary file 1 — Association between clinicopathological data and TFF1 methylation and expression in healthy esophagus. (DOC 129 kb) [file 13148_2017_429_MOESM1_ESM.doc]

**Additional file 1 Table S1: Association between clinicopathological data and *TFF1* methylation and expression in healthy esophagus.**

| **Clinicopathological Data** | ***TFF1* Methylation**  **Median (min – max)** | **p Value** | ***TFF1* mRNA expression**  **2^-ΔCT^ Median (min - ma x )** | **p Value** |
| --- | --- | --- | --- | --- |
| **Gender**  Male  Female | 36.0% (19.8% - 63.0%)  39.1% (25.8% - 57.9%) | p = 0.31 | 1.1 x 10^-3^ (5.13 x 10^-4^ - 1.9 x 10^-2^)  3.0 x 10^-3^ (9.6 x 10^-4^ - 1.8 x 10^-1^) | p = 0.07 |
| **Age**  ≤ Median  > Median | 37.2% (25.8% - 63.0%)  39.1% (19.8% - 57.9%) | p = 0.95 | 2.44 x 10^-3^ (7.4 x 10^-4^ - 1.8 x 10^-1^)  2.1 x 10^-3^ (5.1 x 10^-4^ - 1.5 x 10^-2^) | p = 1.00 |
| **Tobacco Smoking**  Never smokers  Former smokers  Current smokers | 39.1% (19.8% - 57.9%)  39.1% (29.2% - 63.0%)  35.9% (25.8% - 49.8%) | p = 0.83 | 2.6 x 10^-3^ (5.1 x 10^-4^ - 7.3 x 10^-2^)  1.4 x 10^-3^ (7.4 x 10^-4^ - 1.0 x 10^-2^)  1.1 x 10^-2^ (8.2 x 10^-4^ - 1.8 x 10^-1^) | p = 0.30 |
| **Alcohol Drinking**  Never drinkers  Former Drinkers  Current drinkers | 39.2% (19.8% - 57.8%)  53.3% (43.6% - 63.0%)  33.6% (25.8% - 47.9%) | p = 0.05 | 2.5 x 10^-3^ (7.4 x 10^-4^ - 1.6 x 10^-2^)  8.2 x 10^-4^ (7.5 x 10^-4^ - 3.2 x 10^-3^)  2.8 x 10^-3^ (5.1 x 10^-4^ - 1.8 x 10^-1^) | p = 0.13 |
