# Supplementary material for: TFF1 hypermethylation and decreased expression in esophageal squamous cell carcinoma and histologically normal tumor surrounding esophageal cells
Source: Clin Epigenetics. 2017 Dec 20;9:130. doi: 10.1186/s13148-017-0429-0 (PMC5738900; doi:10.1186/s13148-017-0429-0)
Supplement: Supplementary file 2 — Association between clinicopathological data and TFF1 expression in ESCC. (DOC 238 kb) [file 13148_2017_429_MOESM2_ESM.doc]

**Additional file 2: Table S2: Association between clinicopathological data and *TFF1* expression in ESCC.**

| **Clinicopathological Data** | ***TFF1* Methylation in ESCC**  **Median (min - max)** | **p Value** | ***TFF1* mRNA expression in ESCC**  **2^-ΔCT^ Median (min - max)** | **p Value** |
| --- | --- | --- | --- | --- |
| **Gender**  Male  Female | 68.2% (32.6% - 90.2%)  64.1% (29.6% - 85.0%) | p=0.13 | 5.1 x 10^-4^ (1.0 x 10^-6^ – 4.8 x 10^-2^)  2.3 x 10^-4^ (1.3 x 10^-4^ – 3.0 x 10^-4^) | p=0.07 |
| **Age**  ≤ Median  > Median | 62.7% (29.6% - 90.2%)  68.5% (50.3% - 86.3%) | p=0.04 | 4.9 x 10^-4^ (1.0 x 10^-6^ – 3.9 x 10^-3^)  5.3 x 10^-4^ (1.9 x 10^-4^ – 4.8 x 10^-2^) | p=0.39 |
| **Tobacco Smoking**  Never smokers  Former smokers  Current smokers | 66.1% (50.0% - 84.6%)  65.7% (58.6% - 68.9%)  67.5% (29.6% - 90.2%) | p=0.93 | N.A.  3.9 x 10^-4^ (1.0 x 10^-6^ – 7.6 x 10^-4^)  4.5 x 10^-4^ (1.3 x 10^-4^ – 4.8 x 10^-2^) | p=0.96 |
| **Alcohol Drinking**  Never drinkers  Former Drinkers  Current drinkers | 62.9% (29.6% - 84.6%)  76.7% (65.7% - 87.6%)  67.5% (32.6% - 90.2%) | p=0.40 | 2.7 x 10^-4^ (1.0 x 10^-6^ – 5.1 x 10^-4^)  5.7 x 10^-4^ (3.9 x 10^-4^ – 7.4 x 10^-4^)  4.9 x 10^-4^ (1.7 x 10^-4^ – 4.8 x 10^-2^) | p=0.26 |
| **Tumor Differentiation**  Well  Moderate  Poor | N.A.  69.2% (56.7% - 90.2%)  63.8% (59.9% - 67.6%) | p=0.48 | N.A.  4.2 x 10^-4^ (1.0 x 10^-6^ – 3.9 x 10^-3^)  2.4 x 10^-2^ (5.1 x 10^-4^ – 4.8 x 10^-2^) | N.D. |
| **T (TNM)**  T1  T2  T3  T4 | N.A.  N.A.  67.6% (56.7% - 90.2%)  73.7% (59.9% - 87.6%) | p=0.46 | N.A.  N.A.  4.9 x 10^-4^ ( 1.0 x 10^-6^ – 4.8 x 10^-2^)  4.0 x 10^-4^ (2.0 x 10^-4^ – 9.9 x 10^-4^ ) | p=0.81 |
| **N (TNM)**  N0  N1 | 67.6% (64.6% - 68.9%)  65.7% (58.7% - 90.2%) | p=1.00 | 5.7 x 10^-4^ ( 1.0 x 10^-6^ – 4.8 x 10^-2^)  5.4 x 10^-4^ (1.3 x 10^-4^ – 3.9 x 10^-3^) | p=0.88 |
| **M (TNM)**  M0  M1 | 65.7% (58.7% - 90.2%)  81.7% (75.9% - 87.6%) | N.D. | 5.8 x 10^-4^ (1.0 x 10^-6^– 4.8 x 10^-2^)  2.1 x 10^-4^ (1.3 x 10^-4^ – 5.7 x 10^-4^) | p=0.08 |
| **Tumor Stage**  I  II  III + IV | N.A.  67.6% (64.6% - 68.9%)  73.7% (58.7% - 90.2%) | p=0.60 | N.A.  5.5 x 10^-4^ (1.0 x 10^-6^ – 4.8 x 10^-2^)  3.9 x 10^-4^ (1.3 x 10^-4^ – 3.9 x 10^-3^) | p=0.69 |

N.A.: Not available; N.D.: Not determined (n was not enough to perform statistical analysis)
